# Cholecystokinin-B Receptor-Targeted Nanoparticle for Imaging and Detection of Precancerous Lesions in the Pancreas

**DOI:** 10.3390/biom11121766

**Published:** 2021-11-25

**Authors:** Jill P. Smith, Hong Cao, Elijah F. Edmondson, Siva Sai Krishna Dasa, Stephan T. Stern

**Affiliations:** 1 Department of Medicine, Georgetown University, Washington, DC 20007, USA; hc87@georgetown.edu; 2 Molecular Pathology Laboratory, Frederick National Laboratory for Cancer Research, Frederick, MD 21701, USA; elijah.edmondson@nih.gov; 3 Nanotechnology Characterization Laboratory, Cancer Research Technology Program, Frederick National Laboratory for Cancer Research Sponsored by the National Cancer Institute, Frederick, MD 21701, USA; sivasaikrishna.dasa@nih.gov (S.S.K.D.); sternstephan@mail.nih.gov (S.T.S.)

**Keywords:** pancreatic cancer, cholecystokinin receptor, fluorescence, imaging, PanINs

## Abstract

Survival from pancreatic cancer remains extremely poor, in part because this malignancy is not diagnosed in the early stages, and precancerous pancreatic intraepithelial neoplasia (PanIN) lesions are not seen on routine radiographic imaging. Since the cholecystokinin-B receptor (CCK-BR) becomes over-expressed in PanIN lesions, it may serve as a target for early detection. We developed a biodegradable fluorescent polyplex nanoparticle (NP) that selectively targets the CCK-BR. The NP was complexed to a fluorescent oligonucleotide with Alexa Fluor 647 for far-red imaging and to an oligonucleotide conjugated to Alexa Fluor 488 for localization by immunohistochemistry. Fluorescence was detected over the pancreas of five- to ten-month-old LSL-Kras^G12D/+^; *P48-Cre* (KC) mice only after the injection of the receptor target-specific NP and not after injection of untargeted NP. Ex vivo tissue imaging and selective immunohistochemistry confirmed particle localization only to PanIN lesions in the pancreas and not in other organs, supporting the tissue specificity. A human pancreas tissue microarray demonstrated immunoreactivity for the CCK-BR only in the PanIN lesions and not in normal pancreas tissue. The long-term goal would be to develop this imaging tool for screening human subjects at high risk for pancreatic cancer to enable early cancer detection.

## 1. Introduction

The use of nanoparticles (NPs) to improve imaging of tissues and cancers has increased over the past decade since these agents exhibit improved sensitivity, penetration depth, and multi-modal capacity compared to small-molecule imaging agents [1]. Although the uptake of NPs is greater in tumor tissues due to enhanced permeability and retention (EPR), these agents can be functionalized with targeting moieties to render the NPs tissue-specific and decrease off-site uptake and toxicity [2]. For in vivo use, NPs can be designed both as imaging tools for the detection of cancers and also equipped with payloads to deliver therapy at the designated site, i.e., “theranostic” agents [3].

Pancreatic cancer has a dismal prognosis [4,5], and with the current chemotherapeutic regimens, 5-year survival is only about 10% [6]. One reason contributing to the poor outcome of this malignancy is the inability to diagnose pancreatic cancer in early or precancerous stages [4]. The current radiographic imaging tools such as computerized tomography (CT) and magnetic resonance imaging (MRI) lack sensitivity [7] and are limited to detecting tumors greater than 2 cm in size [8]. Endoscopic ultrasound (EUS) is another approach that has been used in the clinic to evaluate pancreatic cysts or intraductal papillary mucinous neoplasms (IPMNs) for pancreatic cancer [9]. However, only about 15% of pancreatic cancers arise from cysts; the majority (85%) of pancreatic cancers develop from a microscopic precursor lesion called pancreatic intraepithelial neoplasia (PanINs). Unfortunately, PanINs are not identified by the standard radiographic imaging or endoscopic techniques.

Early stages of disease leading to the development of pancreatic carcinoma have been difficult to study in human subjects since the majority of those with pancreatic cancer have advanced disease at the time of presentation [10]. A genetically engineered murine model that has conditional expression of an endogenous oncogenic *KRAS* (G12D) allele in the murine embryo has been established [11], and researchers have used variations of this model to study pancreatic carcinogenesis since this model has the same genetic and phenotypic features of human pancreatic cancer. In the current work, we used a variant of this murine model, LSL-Kras^G12D/+^; *P48-Cre*, [12] that highly resembles pathogenesis in the human precancerous pancreas. This murine model progresses through advancing grades of premalignant lesions (PanINs 1, 2, and 3) allowing investigators to study pancreatic carcinogenesis and early-stage malignancy. Since PanIN-3 lesions are considered carcinoma in situ, researchers have been trying to develop tools to facilitate in the detection of these precancerous lesions in order to provide earlier intervention in order to decrease the dismal prognosis of pancreatic cancer. An analogy to the PanIN lesion is that of the adenomatous polyp in the colon that is detected by colonoscopy and removed to prevent colon cancer. If high-grade PanIN lesions could be detected and treated or removed before progressing to pancreatic cancer, the incidence of pancreatic cancer could decrease.

We discovered that a G-protein coupled receptor called the cholecystokinin-B receptor (CCK-BR) is rare in the normal mouse and the normal human pancreas [13], but this receptor becomes over-expressed in PanIN lesions [14] and is markedly over-expressed in pancreatic cancer [15,16]. The major ligand for the CCK-BR, gastrin, is detected in the fetal pancreas [17,18] but is silenced during gestation and not found in the adult pancreas. However, gastrin also becomes re-expressed by microRNA-27a in PanINs and in pancreatic cancer [19], where it stimulates growth by an autocrine mechanism [20]. Downregulation of gastrin mRNA by RNA interference (RNAi) techniques inhibits the growth and metastasis of human pancreatic cancer [21,22]. Hence, mechanisms to deliver RNA interference to silence gastrin expression could have therapeutic potential.

Since gastrin and its receptor, the CCK-BR, become activated in precancerous PanINs and are over-expressed in pancreatic cancer, strategies to target this pathway have been investigated. Using a thiol-maleimide coupling reaction [23], we covalently bound the maleimide functionalized receptor-binding moiety of the gastrin-10 ligand to a thiol-functionalized polyethylene glycol-block-poly (L-lysine) (SH-PEG-PLL) polymer, rendering it target specific to the CCK-BR [24]. The positively charged lysine polymer allowed electrostatic complexation with a negatively charged oligonucleotide to form the stable micelle, while also shielding the positive charge and eliminating toxicity. We previously demonstrated that this receptor-specific polyplex NP was selective in targeting the CCK-BR and delivering gastrin siRNA to successfully inhibit growth and metastases of human pancreatic tumors in mice [24].

Several platforms are being developed that utilize target-specific NPs combined with a metal or dye or both (dual) for imaging in pancreatic cancer [25]. Due to the lack of specific targets, early detection in pancreatic cancer has been problematic. A Plectin-1-targeted dual-modality NP carrying iron oxide has been described for the imaging of orthotopic pancreatic cancer [26]. Another NP that targets galectin-1 combined with Fe_3_O_4_ detected small subcutaneous BxPC-3 human pancreatic tumors [27]. Han et al. [28] developed a gadolinium ion-doped upconversion NP (UCNP) micelle that targets the epithelial cell adhesion molecule (EpCAM, also known as CD326) in pancreatic cancer xenografted to mice. Techniques to improve imaging sensitivity with positron emission tomography (PET) or single photon emission computerized tomography (SPECT) in combination with anatomical techniques, such as computerized tomography (CT), are also being developed. In this context, Benito and colleagues [29] developed a single-chain polymer NP (SCPNs) that targets the somatostatin receptor on pancreatic cancer and loaded the particles with Gallium-67 for SPECT imaging in mice bearing xenografted pancreatic tumors. Unfortunately, all these above studies utilized mice with established pancreatic tumors either implanted orthotopically or subcutaneously and not cancer precursor lesions. Kelly et al. [30] tested a Cy5.5 fluorescent plectin-1-targeted NP crosslinked with iron oxide in mutant KRAS mice with PanIN lesions by making a midline incision over the mouse pancreas and imaging by laser scanning microscopy. Unfortunately, this NP exhibited high biodistribution in the liver and kidney [30] that would potentially limit its clinical utility. Otherwise, there is a paucity of the literature concerning the target-specific imaging of pancreatic PanIN lesions.

Since improving the survival of pancreatic cancer will require early detection or identification of lesions before they become established cancer, the goal of this investigation was to analyze the ability of a fluorescent CCK-BR-targeted NP micelle as an imaging tool to detect precancerous PanIN lesions in vivo in the mutant KRAS mouse pancreas, before pancreatic cancer occurs. Our long-term goal includes the development of this NP for diagnosis and, perhaps in combination with gastrin siRNA or other payloads, for treatment of PanIN lesions and prevention of pancreatic cancer.

## 2. Materials and Methods

### 2.1. Synthesis of the CCK-B Receptor-Targeted Polyplex Nanoparticle

In order to develop the targeted NP, a thiol functionalized polyethylene glycol-block-poly (L-lysine) (SH-PEG-PLL) polymer was synthesized as previously described [24]. In brief, SH-PEG-PLL was synthesized from trityl-S-poly (ethylene glycol)-*block*-poly (L-lysine) (Tr-S-PEG-PLL) (average MW of 9700 g/mol) with PEG MW of 5000 g/mol; Tr-S-PEG-PLL was custom synthesized (Alamanda Polymers, Huntsville, AL, USA) by reducing with trifluoroacetic acid and triethylsilane (98:2 *v*/*v*). To render the NP target-specific for the CCK-BR, we used a maleimide functionalized to the N-terminus of gastrin-10 peptide (Glu-Glu-Glu-Ala-Tyr-Gly-Trip-Met-Asp-Phe-NH_2_, MW 1426.48 g/mole) (custom synthesis by GenScript USA Inc., Piscataway, NJ, USA) to conjugate to thiol group on the polymer (SH-PEG-PLL) via Michael addition reaction at pH 7 in deoxygenated HEPES buffer (100 mM) under an inert atmosphere (Supplementary Figure S1). The resulting Ga-PEG-PLL was extensively purified using a PD-10 column and dialysis (spectrapor^®^ RC membrane, MW cut off 8–10 KD, (ThermoFisher Scientific, Waltham, MA, USA)) and fast phase liquid chromatography with a UV detector set at λ = 220 nm, a size exclusion column (HiPrep 16/60 Sephacryl S-500 HR, GE Healthcare Life sciences, Chicago, IL, USA), and mobile phase consisting of sodium phosphate buffer (pH 7.0 with 0.3 M NaCl), at a flow rate of 1 mL/min. The dual tagged polyplex micelle was prepared by mixing 1 mg/mL of the Ga-PEG-PLL with double stranded 5′ Alexa Fluor 647 and 5′ Alexa Fluor 488 fluorophore labeled oligonucleotides (Life Technologies, ThermoFisher Scientific, Waltham, MA, USA) at an N/P ratio of 5/1 (N/P ratio referring to the molecular ratio of cationic polylysine amines, ‘N’, to anionic RNA phosphates, ‘P’, in the polyplex). The nonspecific sequence of the Stealth Custom RNA, 5′ Alexa Fluor 647 was the following: sense: AGCUACACUAUCGAGCAAUUAACUU and anti-sense: AAGUUAAUUGCUCGAUAGUGUAGCU. The negative Control LOGC_a3N Custom RNA, 5′ Alexa Fluor 488 was proprietary but confirmed by NCBI Blast not to selectively inhibit specific RNA.

### 2.2. Characterization of the Polyplex Micelle Nanoparticle In Vitro 

The self-assembled targeted dual siRNA polyplex nanoparticles were characterized for hydrodynamic size distribution by dynamic light scattering (DLS) using photon correlation spectroscopy with an optical glass round cuvette with a diameter of 5 mm in a 3D LS Spectrometer (LS Instruments, Fribourg, Switzerland), equipped with a HeNe laser at 633 nm (25 °C, 633 nm laser, 90° scattering angle).

Two methods were used to confirm co-assembly of the two fluorophore-labeled siRNAs complexed into the same polyplex micelle. Polyplex stability was accessed by treating with heparin. Heparin sulfates are glycosaminoglycans that are sulfated and have a strong negative charge that can be used to destabilize the electrostatic polyplex. The high negative charge density of heparin results in competitive displacement of siRNA from the polyplex. When run on a Tris-borate-EDTA (TBE) gel (Novex™ 20% TBE gels, catalog# EC63155BOX, ThermoFisher Scientific, Waltham, MA, USA), the neutral, intact polyplex with electrostatically complexed RNA remains in the loading wells at the top of the gel. However, upon RNA displacement from the polyplex by heparin, it is free to migrate into the gel and detected as bands. Dual-tagged RNA polyplex (2 µL of 10 µM siRNA) was added to 13 µL of nuclease free water and mixed with a pipette. A measurement of 5 µL of nuclease free water or heparin solution (15 mg/mL) (Sigma-Aldrich, Inc. St. Louis, MO, USA) was added to siRNA and polyplex and incubated for 10 min at room temperature (RT). Before analyzing the samples on a 20% TBE gel, 5 µL of RNA-loading dye was added to each sample and samples run for 1.5 h at 140 V in a XCell SureLock™ Mini-Cell electrophoresis system (ThermoFisher Scientific, Waltham, MA, USA), with Thermo Electron 3000–90 power supply (ThermoFisher Scientific). To visualize the RNA, the gel was stained with 1% SYBR gold (ThermoFisher Scientific) staining solution for 10 min. Other controls were also included, namely free siRNA with and without heparin, and polymer and heparin only samples. RNA bands were visualized by UV transilluminator of G:BOX gel documentation unit (Syngene, Frederick, MD, USA).

The second procedure to investigate dual complexing of both siRNA fluorophores, Alexa Fluor 488 and Alexa Fluor 647 siRNA, in the same polyplex micelle included analysis of free NP solution in vitro and the incubation with wild-type human PANC-1 pancreatic cancer cells that express the CCK-BR. PANC-1 cells were obtained from ATCC and were cultured in Dulbecco’s modified Eagle medium with 10% fetal bovine serum. Cells were harvested from culture plates and centrifuged to pellet the cells. The cells were washed with phosphate-buffered saline (PBS) and suspended in dye-free Opti-MEM medium (ThermoFisher Scientific, Waltham, MA, USA) and then were plated into each well of 96-well plates at concentrations from 2 × 10^6^, 0.5 × 10^6^, 0.125 × 10^6^, and 0.0625 × 10^6^ cells and no cells. The cells were then incubated in vitro with the dual-complexed polyplex micelle for 1 h. After incubation, the cells were washed to remove any of the free NP, resuspended in PBS, and plated into wells of a 96-well plate. Fluorescence intensity was measured using an IVIS Lumina III in Vivo Optical Imaging System IVIS (Perkin Elmer, Waltham, MA, USA) with images of the same cells acquired for Alexa Fluor 488 at (460–520; excitation/emission) and for Alexa Fluor 647 at (620–670; excitation/emission) in order to determine the fluorescence in the cells of each fluorophore independently.

### 2.3. Analysis of the CCK-B Receptor as a Target for the Polyplex Nanoparticle

Wild-type (WT) human PANC-1 cells that express the CCK-BR [16,31] and PANC-1 cells that were stably transfected to over-express the CCK-BR (CCK-BR-OE) as described [32] were utilized to investigate the binding capacity of untargeted polyplex micelle or CCK-BR target-specific polyplex. PANC-1 WT and CCK-BR-OE cells were harvested, centrifuged, washed, and suspended in Opti-MEM medium. Cells were suspended in each well of 24-well plate at six various cellular concentrations ranging from 10^6^ cells to 3.1 × 10^4^ cells and then were incubated with either the targeted or untargeted dual Alexa-fluorophore polyplex micelle for 1 h in vitro. After the incubation period, cells were removed and washed in PBS to remove free fluorescent NPs. Each prospective treated cell group (10^6^ cells to 3.1 × 10^4^) was placed in a 96-well plate and imaged in the IVIS machine. PBS solution only was used as a negative control. Fluorescence of the cells in the far-red range was captured for each group of cells (targeted versus untargeted) and wild type versus CCK-BR-OE PANC-1 cells in the IVIS System at fluorescence 647 (620–670).

### 2.4. Breeding and Genotyping of Mice 

The mouse genomic KRAS locus was upstream of a modified exon 1 engineered to contain a c.35G>A nucleotide change resulting in a glycine to aspartate transition (G > D) in codon 12. This mutation is commonly found in human pancreatic adenocarcinoma, and expression of the mutated allele is achieved by interbreeding LSL-Kras^G12D^ mice with animals that express Cre recombinase from the pancreatic-specific promoter, P48. LSL-Kras^G12D/+^; *P48-Cre* (*KC*) mice were bred and genotyped as previously described [12]. Heterozygote breeders (male or female) were mated. This Kras allele is non-functional in its germline configuration; therefore, the mice are maintained by backcrossing heterozygous animals to C57BL/6. The usual litter size is approximately 8. At the time of weaning, mouse genotypes were determined by PCR analysis of tail DNA preparations. Tail biopsies were obtained from <3-week-old mice with <0.5 cm removed after topical application of ice cold ethanol for anesthesia. Approximately 1:4 pups will be genotype LSL-Kras^G12D/+^; *P48-Cre* and develop PanINs. Hence, approximately 136 mice were genotyped for us to have the *N* = 34 used in this investigation.

### 2.5. Administration and Imaging of KC Mice and Organs with Polyplex Nanoparticles

All animal studies were performed in an ethical fashion under a protocol approved by the Georgetown University IACUC. In this transgenic model, PanINs begin to develop by 3 months and PanIN-3 lesions by 4–6 months, and cancer can occur by 8–10 months. The rationale for these cohorts is that by an age of 5 months, the mice with this LSL-Kras^G12D/+^; *P48-Cre* genotype [12] will have developed PanINs of all three stages (PanIN 1, 2, and 3), but rarely pancreatic cancer. By an age of 10 months, early pancreatic cancer may be found histologically. Ten days before performing imaging in the IVIS System, mice were changed to purified alfalfa-free diet (ENVIGO, cat# TD.97184; Indianapolis, IN, USA) to decrease any auto-fluorescence from food in the far-red range. The fur from the mouse abdomen and mid back was shaved prior to imaging. The optimal time to harvest organs after injection of the polyplex micelle was determined by evaluating the fluorescent emission or fluorescent emission in the IVIS System in mice that ranged from 5–8 months of age. Mice were anesthetized with isoflurane and a baseline image was obtained using the IVIS Lumina III In Vivo Optical Imaging System. After baseline images were recorded, mice were injected with the CCK-BR target-specific NP in a 0.1 mL volume via an intraperitoneal injection, and fluorescent emission was measured with the epi-fluorescent 620–670 filter at 3, 4, 5, and 6 h and again 24 h after injection. Each mouse was injected 2–3 times over the period of one week after, allowing 24–48 h for clearance, and (*N* = 12) mice were used for the uptake experiment. The peak fluorescent emission was determined to be 5 h, and this time interval was selected to harvest organs for ex vivo imaging in mice injected with targeted or untargeted NPs. Mice used for the ex vivo experiments and immunohistochemistry included mice of 4, 5, 6, 7, 8, and 10 months of age (*N* = 16 mice). For controls, age-matched wild type C57BL/6 mice were injected with the targeted polyplex NP (*N* = 3), and age-matched 5-month-old, 7-month old, and 8-month-old LSL-Kras^G12D/+^; *P48-Cre* mice were injected with untargeted polyplex (*N* = 3 per group). All polyplex NPs (whether targeted or non-targeted) injected were complexed with both the Alexa Fluor 488-labeled siRNA for immunohistochemistry (IHC) and Alexa Fluor 647-labeled siRNA for imaging.

Since 5 h was identified as the time of peak fluorescence after polyplex injection, this time was also selected for harvesting of the pancreas and other major organs histological analysis. Tissues were excised and imaged ex vivo in the IVIS System to compare targeted versus untargeted treated mice, followed by tissue fixation with 4% paraformaldehyde at room temperature for 18–24 h and paraffin embedding.

### 2.6. Immunohistochemistry for Detection of Alexa Fluor 488 Labeled Polyplex Nanoparticles in Tissues 

Immunohistochemistry to detect Alexa Fluor 488 was performed using an ImmPRESS^®^ HRP Horse Anti-Rabbit IgG PLUS Polymer Kit, Peroxidase (Vector Labs, Burlingame, CA, USA, Catalog #: MP-7451) on 5 μm tissue sections mounted on Fisherbrand™ Superfrost™ Plus Microscope Slides (ThermoFisher Scientific), which were dewaxed and rehydrated with double-distilled H_2_O. Heat-induced epitope retrieval (HIER) was performed by heating sections in 0.01% citraconic anhydride containing 0.05% Tween-20 in a pressure cooker set at 122–125 °C for 30 s. Slides were incubated with blocking buffer (TBS with 0.05% Tween-20 and 0.25% casein) for 10 min and then incubated with rabbit anti-Alexa Fluor 488 antibody (1:400; Cat. No. A-11094, Invitrogen) diluted in blocking buffer over night at 4 °C. Slides were washed in 1X TBS with 0.05% Tween-20 and endogenous peroxidases blocked using 1.5% (*v*/*v*) H_2_O_2_ in TBS (pH 7.4) for 10 min. Slides were incubated with Rabbit Polink-1 HRP (Vector Labs) for 30 min at room temperature, washed, and incubated with Impact™ DAB (3,3′-diaminobenzidine; Vector Laboratories) for 2–5 min. Slides were washed in ddH_2_O, counterstained with hematoxylin, and mounted in Permount (ThermoFisher Scientific). Whole tissue sections were scanned at high magnification (200X) using the ScanScope AT2 System (Leica Biosystems, Buffalo Grove, IL, USA), yielding high-resolution data from the entire tissue section. Representative high-resolution images were extracted from these whole-tissue scans. Confirmatory immunohistochemical stains were performed with a rabbit monoclonal anti-PEG (Cat#ab51257; Abcam; Waltham, MA, USA) diluted at 1:1000 following heat-induced epitope retrieval in citrate buffer for 20 min.

### 2.7. Immunohistochemistry (IHC) for CCK-BR in KRAS Mouse Pancreas and Human Pancreas

In order to confirm that the mouse PanINs that accumulated the CCK-BR-targeted NP indeed expressed CCK-BR, CCK-BR immunohistochemistry (IHC) was performed on tissue sections (5 µm) from 5-month-old mutant KRAS mouse pancreas. To investigate the clinical importance of our findings and the potential for this CCK-BR target-specific polyplex NP to be used as an imaging tool for early detection of high grade PanINs or pancreatic cancer in human subjects, CCK-BR IHC was also performed on a human pancreas tissue microarray (TMA) obtained from US Biomax, (Rockville, MD, USA, Cat. No. BIC14011b). The human pancreas tissue microarray contained 48 unstained cores of fresh frozen paraffin-embedded human pancreas tissues from normal pancreas obtained at autopsy and from subjects with various grades of PanINs. Of these, there were *N* = 5 subjects with PanIN grade 1, *N* = 6 with PanIN grade 2, *N* = 4 with only PanIN grade 3, and *N* = 8 with both PanIN grade 3 and cancer (Total = 12), and there were *N* = 8 sections from normal controls. The KRAS mouse pancreas tissue sections and the human tissue microarray were deparaffinized and subjected to antigen retrieval. The slides were washed in 1× PBS 3× for 2 min, and then blocking was performed according to the manufacturer instructions (Anti-goat HRP-DAB IHC Detection Kit; CTS008-NOV, Novus Biologicals, Centennial, CO, USA). The slide was then incubated with the primary antibody CCK-BR (Cat#Ab77077, Abcam) at 1:200 titer in PBS overnight at 4 °C. After rinsing, the slide was incubated with 1–3 drops of Biotinylated Secondary Antibody (Novus Biologicals) for 60 min. The slide was then treated with 1–3 drops of High Sensitivity Streptavidin conjugated to horse radish peroxidase (HSS-HRP) (Novus Biologicals) for 30 min and washed. Visualization was achieved by enzymatic conversion of the chromogenic substrate 3,3′ Diaminobenzidine (DAB) into a brown-colored precipitate by horseradish peroxidase (HRP) at the sites of CCK-BR localization. Images were scanned using an Aperio GT450 automated (Leica Biosystems, Buffalo Graove, IL, USA), high-capacity digital pathology slide scanner and images captured with software from Aperio Image Scope. The images were analyzed for intensity of CCK-BR staining using the public domain software ImageJ (NIH Image, Bethesda, MD, USA) and corrected for area of tissue examined.

### 2.8. Statistical Analysis

For immunohistochemical comparisons between normal mouse pancreas tissues and PanINs, images were scanned using an Aperio GT450 machine and images captured (*N* = 10 each grade) with software from Aperio Image Scope. CCK-BR IHC was analyzed by densitometry with Image-J software corrected for area of tissue examined. Statistical analysis was performed with PRISM GraphPad software with Bonferroni correction applied for multiple comparisons to control tissue. Fluorescent emission of PANC-1 cells were recorded by the IVIS instrument, and mean emission intensity values were normalized for each wavelength with emission intensity at 2 million cells as 100% and zero cells at 0 for each of the 647 and 488 wavelengths. This normalized the mean values for the fluorophore fluorescence efficiency and allowed direct comparison of the fluorescent emission/cell density response of both fluorophores.

## 3. Results and Discussion

### 3.1. Synthesis of the CCK-B Receptor-Targeted Polyplex Nanoparticle

**Figure 1:**
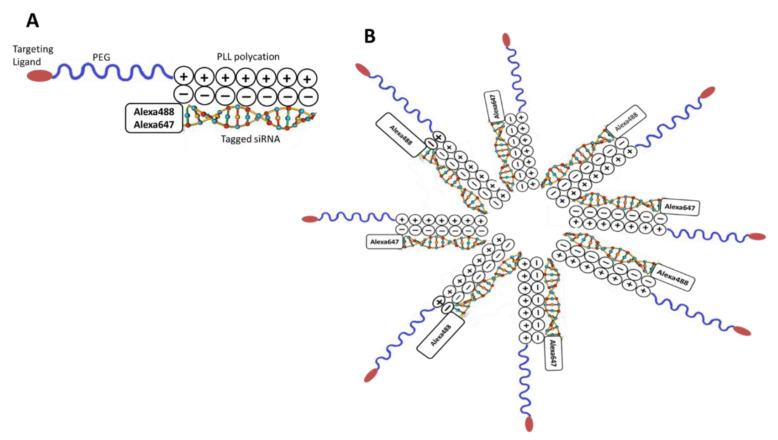
Formulation of the CCK-BR target-specific polyplex NP micelle. (**A**) The gastrin-10 targeting ligand moiety is conjugated to polyethylene glycol (PEG), which in turn is conjugated to a poly L-lysine block chain rendering the backbone positively charged. The oligonucleotides were conjugated with either Alexa Fluor 488 or Alexa Fluor 647. (**B**) When the positively charged L-lysine co-polymer block complexed with the negatively charged fluorophore-tagged oligonucleotide, a self-assembled polyplex micelle formed.

A polyplex micelle NP was developed to selectively target the CCK-BR expressed on PanIN lesions in the pancreas during pancreatic carcinogenesis [24] from a thiol-functionalized polyethylene glycol-block-poly (L-lysine) (SH-PEG-PLL). The backbone was rendered specific to the CCK-BR by the conjugation of gastrin-10 peptide (Ga-10) to the polymer by a thiol–maleimide coupling reaction (Figure 1A). When the positively charged lysine backbone moiety of this NP was electrostatically complexed with negatively charged oligonucleotides, a self-assembled polyplex micelle formed (Figure 1B). For imaging purposes, the polyplex NP was complexed simultaneously with two separate oligonucleotides: custom RNA, 5′ Alexa Fluor 647 and 5′ Alexa Fluor 488, each at a final concentration of 480 nM. The Alexa Fluor 647-tagged RNA was selected for its fluorescent properties in the far-red wavelength range that allowed in vivo imaging of mutant KRAS mice with an IVIS Lumina III In Vivo Optical Imaging System for biodistribution evaluation. The Alexa Fluor 488-tagged RNA was also used in the polyplex micelle so that high resolution localization of the polyplex micelle in early PanIN lesions in the pancreas and other organs could be confirmed ex vivo by co-registration of anti-Alexa Fluor 488 immunohistochemistry and histopathology (i.e., H&E) images.

### 3.2. Characterization of the Polyplex Micelle Nanoparticle In Vitro

**Figure 2:**
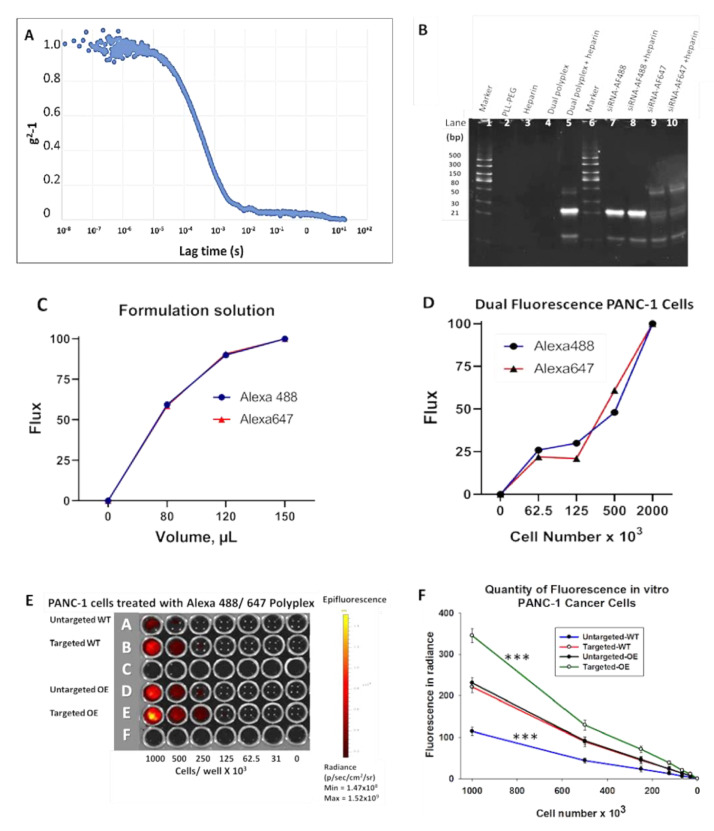
Characterization of the dual fluorophore siRNA polyplex micelle in vitro. (**A**) The dual tagged polyplex NPs were characterized for their hydrodynamic radius by dynamic light scattering (DLS) using photon correlation spectroscopy (PCS). The graph of the electric field autocorrelation function of scattered light from dual nanoparticles of size 91.58 nm and the corresponding fit to the data using single exponential decay is shown. Experiment was performed; (*N* = 3) (**B**) Heparin displacement assay. The polyplex sample was treated with and without heparin and samples run on 20% TBE gel to visualize siRNA release. Without heparin treatment, the polyplex was stable, and there was no free siRNA (Lane 4), but with heparin treatment, siRNAs were released from polyplex (Lane 5). Individual siRNAs (Alexa Fluor 488 and Alexa Fluor 647 tagged) alone when treated with heparin were not affected (Lanes 7–10). (M represents molecular weight marker, AF488 represents Alexa Fluor 488, and AF647 represents Alexa Fluor 647.) (**C**) Fluorescent emission of dual tagged NP solution imaged at the two wavelengths demonstrated comparable fluorescent emission curves at increasing volumes for both the Alexa Fluor 488 and Alex Fluor 647 fluorophores when normalized for fluorescence efficiency of the fluorophores; (*N* = 2) (**D**) Human PANC-1 cancer cells treated with the targeted dual siRNA fluorophore NPs and measured at 488 and 647 wavelengths. The number of cells treated in each well is shown on the X-axis in increasing concentration. The percent of fluorescent emission normalized to 100 is also shown for each wavelength; (*N* = 4). Not significant at all points between Alexa Fluor 488 and 647. (**E**) Wild-type (WT) PANC-1 cancer cells (rows A and B) were treated with untargeted or targeted polyplex, and fluorescence was measured in the IVIS machine. PANC-1 cells that over-express (OE) the CCK-BR that were treated with untargeted (row D) or targeted (row E) polyplex micelle NP are shown with decreasing number of cells per well from left to right. The cell number per well is recorded below the figure and the fluorescent emission intensity scale is shown on the right. (**F**) Mean fluorescence ± SEM from replicate samples including Figure 2E is plotted to demonstrate that the CCK-BR over-expressing PANC-1 cells treated with the targeted polyplex have the greatest fluorescent emission, and the untargeted treated WT PANC-1 cells have the lowest fluorescent emission. (*N* = 2); *** Significantly different comparing targeted to untargeted NPs and significantly different comparing PANC-1 CCK-BR OE targeted cells to both groups or wild-type cells; *p* < 0.001.

After self-assembly, the dual tagged polyplex NPs were characterized for their hydrodynamic radius by dynamic light scattering (DLS) using photon correlation spectroscopy (PCS) as described [33]. Each measurement was conducted in triplicate with a laser at a wavelength of 632.8 nm and a scattering angle of 90° for 20 s with the mean size distribution of 91.58 nm for the targeted polyplex micelle (Figure 2A). The polydispersity index (PDI) of the NP was ~0.32.

#### 3.2.1. Heparin Displacement Assay

A series of in vitro experiments were performed in order to confirm the self-assembly of this micelle NP and that the Alexa Fluor 647- and Alexa Fluor 488-tagged siRNA are co-assembled in the same polyplex. The principal of the heparin displacement assay is that negatively charged heparin is able to displace negatively charged dsRNA from the electrostatic complex with positively charged polylysine, resulting in a release of the complexed dsRNA. When run on a TBE gel, the neutral, intact polyplex with electrostatically complexed RNA remains in the loading wells at the top of the gel. However, upon RNA displacement from the polyplex by heparin, it is free to migrate into the gel and detected as bands. From the heparin displacement assay, it is evident that there is polyplex formation with inclusion of both fluorophore-tagged siRNAs (Figure 2B, lane 4). The absence of any RNA band in the polyplex sample without heparin suggests that polyplex is intact and contains no free RNA. When polyplex is disrupted by heparin treatment, it releases both RNAs that are visualized on the gel (Figure 2B, lane 5). Heparin and polymer-alone control samples did not stain with SYBR gold staining solution, as expected. Individual RNAs with and without heparin treatment showed the same bands. Note that the Alexa Fluor 488-labeled RNA fluoresces brightly in the UV transilluminator, and both single and double stranded RNA are observed, as well as some contaminating untagged RNA species from the tagged RNA synthesis, as is commonly observed. Based on the results of the heparin displacement assay, as expected, there is no free RNA in the polyplex sample. Therefore, all RNA added to form the polyplex is in the polyplex micelle (i.e., a concentration of 480 nM each).

#### 3.2.2. Confirmation of Dual Fluorophore Labeling In Vitro

Fluorescence emission was measured in the IVIS instrument using either the epi-fluorescent 460–520 filter for the measurement of Alexa Fluor 488 or the epi-fluorescent filter of 620–670 for the measurement of Alexa Fluor 647. The fluorescent emission of dual tagged polyplex NP solution imaged in the IVIS instrument demonstrated comparable fluorescent emission curves at increasing volumes when normalized for fluorescence efficiency of the fluorophores (Figure 2C). The uptake of the dual tagged polyplex NP was also performed in human PANC-1 pancreatic cancer cells to investigate the uptake of the polyplex micelle into cells and the micelle Alexa Fluor 647/Alexa Fluor 488-tagged siRNA co-assembly, with equal distribution of both Alexa Fluor 647 and Alexa Fluor 488 siRNAs. Figure 2D reveals the normalized signal data from PANC-1 cells treated with the complexed micelle NP at various cell densities in vitro imaged in the IVIS at the 488 and 647 wavelengths. The intensity of the 488 and 647 fluorescent emission increases with the number of cells, demonstrating equivalent distribution of the Alexa Fluor 647-/Alexa Fluor 488-tagged siRNA in the formulated polyplex. These cells were washed to remove any free polyplex; thus, any fluorescence recorded was from intracellular uptake. These data support that there is equal complexing of the dual siRNA fluorophores in the NP. Furthermore, the normalized signal data demonstrate an equivalent fluorescent emission/cell density response for both fluorophores.

#### 3.2.3. CCK-B Receptor-Targeted Nanoparticles Have Enhanced Uptake in Cells

In order to demonstrate that selective targeting of the CCK-BR in pancreatic cancer improves uptake of the polyplex, we examined the fluorescent emission of wild type human PANC-1 cells and PANC-1 cells engineered to over-express the CCK-BR using NPs that were untargeted or targeted and using the epi-fluorescent filter of 620–670 for measurement of Alexa Fluor 647. Treating CCK-BR-expressing wild-type PANC-1 cells with the targeted polyplex micelle enhances uptake in comparison to the untargeted NPs (Figure 2E; rows A and B). We show in our previous experiments with the CCR-BR-targeted polyplex that the construct is internalized, based on gene knockdown and confocal microscopy studies with fluorescently tagged polyplex [24]. PANC-1 cells transfected to over-express (OE) the CCK-BR (Figure 2E, rows D and E) have a marked increase in the uptake of the targeted polyplex NP compared to the same density of wild-type cells (Figure 2E; rows A and B). Targeting the polyplex micelle to CCK-BR enhances uptake of the NPs, as exhibited by the heightened fluorescent emission compared to the over-expressing cells treated with the untargeted NP. The mean ± SEM fluorescence is plotted for replicate treatments in wild-type and in CCK-BR over-expressing cells in Figure 2F, showing that the untargeted wild-type cells have the least fluorescence, and the targeted over-expressing PANC-1 cells have the greatest fluorescence. Furthermore, the mean fluorescence was significantly increased in the PANC-1 cells treated with the targeted NPs compared to the untargeted NPs (*p* < 0.001).

### 3.3. In Vivo Imaging of KC Mice with Targeted Fluorescent-Tagged Nanoparticles

**Figure 3:**
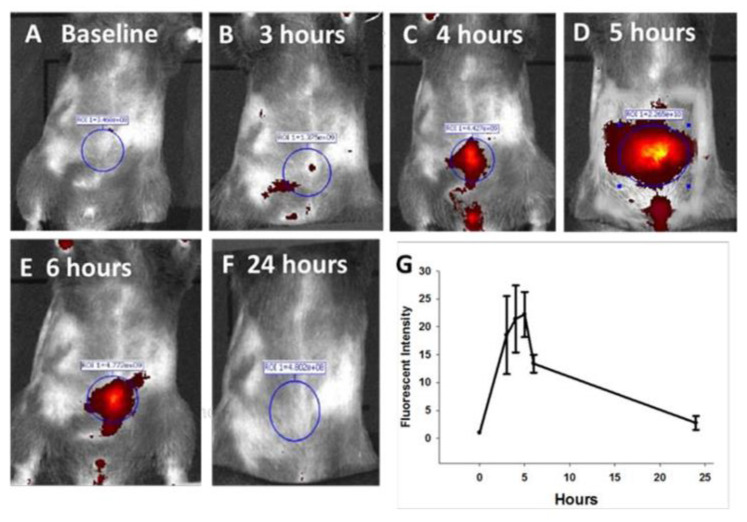
In vivo Alexa Fluor 647 imaging of LSL-Kras^G12D/+^; *P48-Cre* (KC) mice post IP injection of Alexa Fluor 647/ 488-labeled polyplex NP. (**A**–**F**) In vivo image of 5-month-old anesthetized KRAS mouse in the IVIS instrument is shown over time after IP injection of targeted dual polyplex nanoparticle and imaged in the far-red range. Images include (**A**) baseline and post-injection images using epi-fluorescent 620–670 filter at (**B**) 3 h, (**C**) 4 h, (**D**) 5 h, (**E**) 6 h, and (**F**) 24 h. (**G**) Plotted fluorescent emission from 12 different age-matched mice shows mean ± SEM fluorescent emission recorded in IVIS at baseline and at 3, 4, 5, 6, and 24 h post-injection of targeted dual polyplex NP.

LSL-Kras^G12D/+^; *P48-Cre* (*KC*) mice from our genetically engineered animal colony were selected at ages ranging from four months of age, an age when high grade PanIN lesions begin to develop histologically [34], and up to ten months of age, when most PanIN lesions are typically grade 3 or carcinoma in situ. An initial experiment with sequential imaging over a 24 h period was performed to determine the optimal time for NP uptake in the pancreas after injection. Fluorescence in the pancreas was only visualized in the mice treated with the CCK-BR target-specific fluorescent polyplex NP. Images of a representative 5-month-old anesthetized mouse are shown at baseline and 3, 4, 5, 6, and 24 h post IP injection (Figure 3A–F) within the IVIS imaging system, demonstrating fluorescence in the far infrared range consistent with uptake of the Alexa Fluor 647-labeled polyplex localized in the area of the pancreas. The mean ± SEM for age-matched mice is plotted over time (Figure 3G), demonstrating that peak fluorescent intensity in the pancreas was reached 5 h after injection and was absent 24 h after injection. Of note, we previously found that 5 h was also the peak NP uptake in mice bearing orthotopic human pancreatic tumors using NPs tagged with the fluorophore Cy3 [24]. Therefore, this 5 h time point was selected to ethically euthanize the mice after treatment and collect the pancreas and other organs to examine ex vivo for fluorescence and histology. With the same gain settings as the images acquired by IVIS in Figure 3 for the targeted construct, a representative 5-month-old KC mouse injected with untargeted NPs shows no specific, high intensity fluorescence in the mouse pancreas at 5 h post dose (Supplementary Figure S2).

### 3.4. Ex Vivo imaging of Organs from Mice Injected with Targeted or Untargeted Nanoparticles

**Figure 4:**
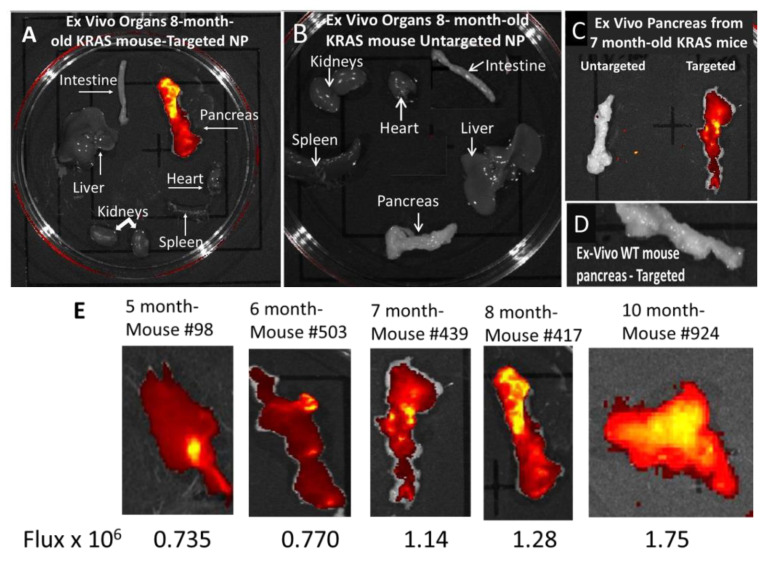
Ex vivo Alexa Fluor 647 imaging of mouse organs after injection of fluorescent nanoparticles. (**A**) Ex vivo fluorescent imaging (epi-fluorescent 620–670 filter) of major organs removed at 5 h post-injection with CCK-BR-targeted fluorescent dual polyplex nanoparticle in an 8-month-old KRAS mouse shows only significant fluorescence in the pancreas. (**B**) Ex vivo fluorescent imaging (epi-fluorescent 620–670 filter) of major organs removed at 5 h post-injection with untargeted fluorescent dual polyplex nanoparticle in an age-matched 8-month-old KRAS mouse shows no fluorescence in the organs. (**C**) Ex vivo fluorescent imaging (epi-fluorescent 620–670 filter) of pancreata removed from 7-month old KRAS mice injected with either untargeted dual fluorescent polyplex (left) or targeted dual fluorescent polyplex NPs (right). (**D**) Ex vivo fluorescent imaging (epi-fluorescent 620–670 filter) of pancreas from a wild-type C57BL/6 control mouse injected with targeted dual polyplex nanoparticles shows no fluorescence. (**E**) Fluorescent emission (Flux) is shown in representative pancreata from mice of increasing age excised 5 h post injection with targeted NPs.

Organs excised at 5 h from an 8-month-old KRAS mouse treated with CCK-BR-targeted polyplex NPs are shown in Figure 4A. Only the excised pancreas demonstrated positive fluorescence ex vivo in comparison to the other organs (Figure 4A). Ex vivo organs harvested from an age-matched KRAS mouse 5 h after being injected with untargeted nanoparticles do not reveal any fluorescence (Figure 4B). Ex vivo pancreata excised from 7-month-old KRAS mice are shown side-by-side (Figure 4C), demonstrating in another aged-matched group that the pancreas is fluorescent only in the mouse treated with the targeted NPs. Note that as the mouse increases in age (comparing the pancreas of the 8-month-old mouse, Figure 4A, to that of the 7-month-old mouse, Figure 4C), the fluorescence in the pancreas increases, correlating with the increased number of PanIN-3 lesions. There was no evidence of fluorescence identified in the pancreas of wild-type control mice injected with the targeted polyplex NP linked with the same fluorescent oligonucleotide probes (Figure 4D). A representative mouse pancreas and fluorescent emission are shown for mice aged 5, 6, 7, 8, and 10 months (Figure 4E). The intensity after NP injection increases in the ex vivo pancreas with the age of the mouse and corresponds to the increasing grade of PanINs.

### 3.5. Confirmation of Nanoparticle Uptake in PanINs by Immunohistochemistry

**Figure 5:**
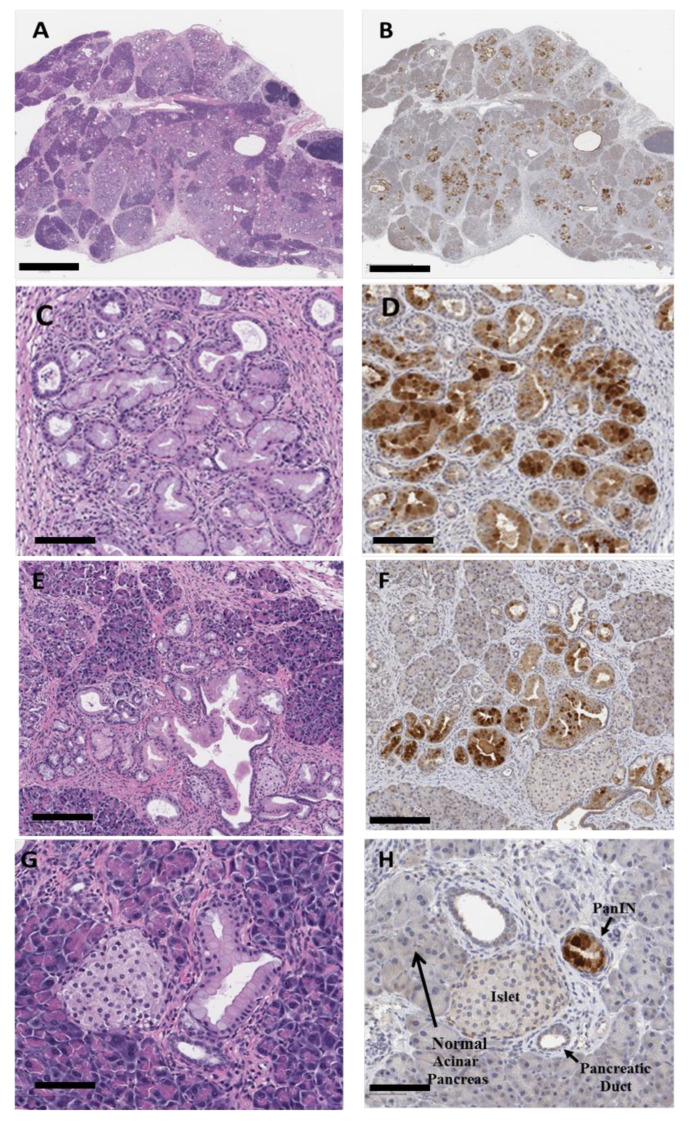
Histology and anti-Alexa Fluor 488 immunohistochemistry of LSL-Kras^G12D/+^; *P48-Cre* murine pancreas. (**A**) H&E histologic section of the 10-month-old mouse pancreas reveals evidence of advanced PanIN lesions and fibrosis of the pancreas microenvironment. Bar = 1 mm. (**B**) The same pancreas section probed with anti-Alexa Fluor 488 antibody shows marked immunoreactivity consistent with the CCK-BR-targeted polyplex micelle NP localization in advanced PanIN lesions. Bar = 1 mm. (**C**) Higher magnification H&E histologic section of 10-month-old mouse pancreas. Bar = 100 μm. (**D**) The same 10-month-old mouse pancreas section probed with anti-Alexa Fluor 488 antibody shows marked immunoreactivity consistent with the targeted polyplex micelle NP localization in advanced PanIN lesions. Bar = 100 μm. (**E**) H&E histologic section of 5-month-old mouse pancreas. Bar = 100 μm. (**F**) Anti-Alexa Fluor 488 IHC shows that the intensity of the anti-Alexa Fluor 488 antibody staining is greatest in the PanIN-3 lesions in the pancreas of the 5-month-old mouse. The normal pancreatic acinar cells are void of staining. Bar = 100 µm. (**G**) H&E at higher magnification of 5-month-old mouse. Bar = 200 μm. (**H**) Selective anti-Alexa Fluor 488 immunoreactivity with intense staining in PanIN-3 lesions of the pancreas of a 5-month-old KC mouse. Arrow on left shows unstained normal pancreatic acinar cells, and small arrow at bottom shows normal ducts. Bar = 200 µm.

In order to confirm that the fluorescent-labeled targeted polyplex NPs accumulated in CCK-BR-expressing pancreatic PanIN lesions and that there was limited off-target uptake in other organs, excised tissues were fixed and paraffin-embedded for immunohistochemistry (IHC) and hematoxylin and eosin (H&E) analysis. Immunochemistry analysis of Alexa Fluor 488 was chosen instead of fluorescence as it allows for the high-resolution correlation of polyplex distribution and H&E histological lesion grade in order to validate CCR-BR as a biomarker/target for early PanIN detection by the targeted polyplex. Fluorescence data, while supportive, would not have been as compelling for this purpose. Tissue sections from the pancreas and other major organs were examined by IHC with a selective rabbit anti-Alexa Fluor 488 antibody and then visualized with Rabbit Polink-1 horseradish peroxidase (HRP) staining. A representative low power image of a 10-month-old mouse pancreas stained with H&E revealed the characteristic pancreatic histology, with advanced PanIN lesions and surrounding fibrosis that occurs during pancreatic carcinogenesis (Figure 5A). Confirmation that the CCK-BR-targeted polyplex micelle NPs were distributed to the high grade PanIN lesions was demonstrated by IHC in the pancreatic tissue section stained with a selective Alexa Fluor 488 antibody (Figure 5B). Higher magnification of this 10-month-old mouse pancreas stained with H&E shows high grade PanIN-3 lesions (Figure 5C). The corresponding tissue shown in Figure 5D stained with anti-Alexa Fluor 488 shows that the immunoreactivity was more intense in the high grade PanIN-3 lesions, with minimal staining in the earlier stage PanIN-1b and PanIN 2 lesions and an absence of staining in the normal pancreatic acinar cells. H&E stain of a KRAS pancreas from a 5-month-old mouse is shown at low magnification (Figure 5E) and at higher magnification (Figure 5G). The same tissues from the 5- month-old mice demonstrate positive immunoreactivity for Alexa Fluor 488 localizing to the high grade PanINs in Figure 5F,H, respectively. Note the lack of staining in the normal pancreatic acinar cells and islet cells of the pancreas, confirming that the NP micelle is selectively targeting the PanIN epithelial cells that express the CCK-BR. This immunoreactivity and the intense fluorescence in the ex vivo pancreas confirm that the fluorescence identified in the living mice 5 h after intraperitoneal injection of the dual Alexa Fluor 488-/647-labeled polyplex NP was indeed the visualization of polyplex within the PanIN lesions of the mouse pancreas. These data are proof of principle that the CCK-BR-targeted NPs reach the mouse pancreas and are taken up into the abnormal precancerous epithelium 5 h after injection.

### 3.6. Immunoreactivity with Anti-PEG and Anti-CCK-BR Antibodies in 5-Month-Old KRAS Mouse Pancreas

**Figure 6:**
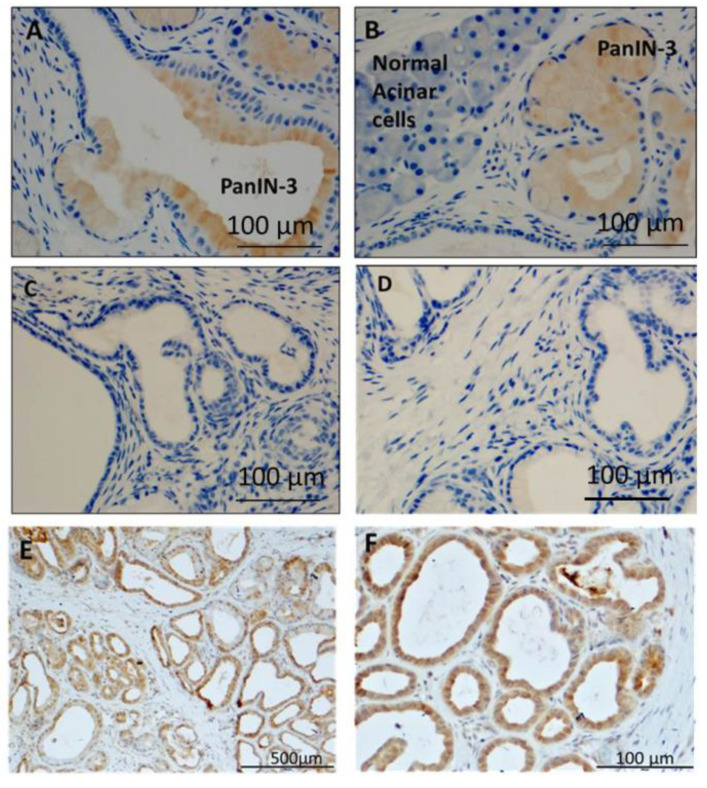
Immunoreactivity with anti-PEG and anti-CCK-BR antibodies in 5-month-old *KRAS* mouse pancreas. (**A**) Pancreas from a mouse treated with targeted polyplex NPs shows PEG staining in the PanINs. (**B**) A PanIN lesion in pancreas of a 5-month-old mouse treated with targeted polyplex NPs shows immunoreactivity for PEG, while adjacent normal acinar cells in pancreas tissue are negative. (**C**) Negative immunoreactivity to PEG antibody in PanINs of 5-month-old *KRAS* mice injected with untargeted polyplex nanoparticles. (**D**) Image from a 5-month-old KRAS mouse pancreas injected with untargeted polyplex nanoparticles is negative for immunoreactivity for PEG. (**E**) KRAS mouse pancreas reacted with CCK-BR goat polyclonal antibody (1:200; Cat# 77077; Abcam) shows selective immunoreactivity in the PanIN epithelial cells. (**F**) Higher magnification of KRAS mouse pancreas stained with a CCK-BR antibody shows selective staining in the PanIN lesions.

Localization of the CCK-BR-targeted polyplex NP in the pancreatic PanINs of mice by immunoreactivity to polyplex components was further confirmed with an antibody to polyethylene glycol (PEG). Similar to the immunohistochemical localization with the Alexa Fluor 488 antibody above, the PEG antibody showed increased staining in the mouse pancreas high grade PanIN lesions (Figure 6A,B). The normal pancreatic acinar cells that lack the CCK-BR did not exhibit any immunoreactivity to the PEG antibody (Figure 6B). Age-matched KC mice injected with untargeted polyplex NP and probed with the same PEG antibody did not reveal any immunoreactivity in the pancreas PanIN lesions (Figure 6C,D). Mouse pancreas from the KC mice that reacted with a CCK-BR selective antibody demonstrated CCK-BR expression in the epithelial cells of the high grade PanIN lesions (Figure 6E,F). These findings are confirmatory evidence that the fluorescence observed in the anesthetized mice by the IVIS imaging system upon targeted polyplex NP injection was indeed due to uptake of the NP in the CCK-BR-expressing precancerous PanIN lesions of the mouse pancreas. Furthermore, these findings support the specificity of the CCK-BR-targeted NP for PanIN tissue uptake and localization and the lack of specificity with the untargeted NP.

### 3.7. The Targeted Polyplex Nanoparticles Has Limited Off-Target Toxicity to Other Organs

**Figure 7:**
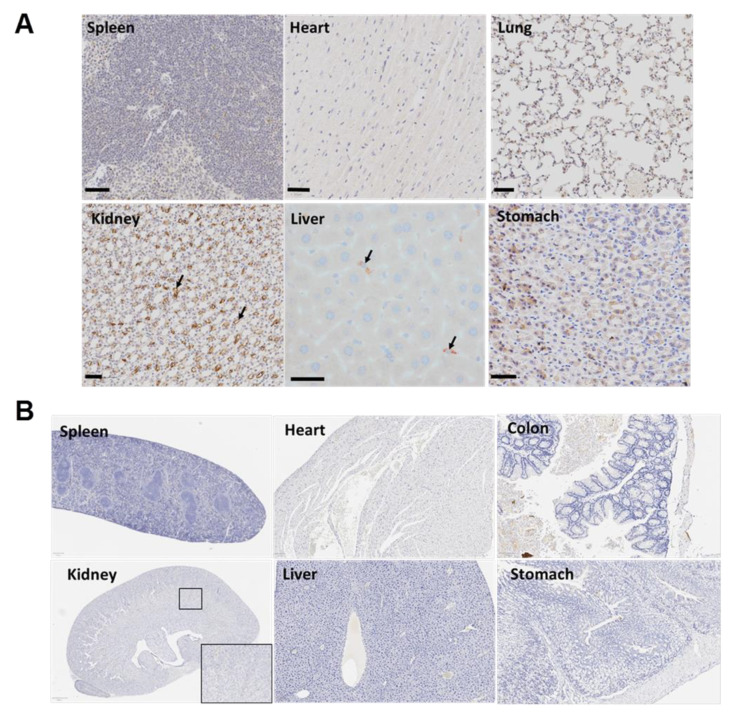
Immunohistochemistry with Alexa Fluor 488 and PEG antibodies in various organs of mouse after injection of the CCK-BR-targeted polyplex micelle NP. (**A**) Immunoreactivity is negligible in other organs stained with the Alexa Fluor 488 antibody. Some brown staining is seen in the kidney in tissues reacted with the Alexa Fluor 488 antibody due to fluorophore excretion in the renal tubules. (**B**) Ex vivo organs of a mouse treated with the targeted dual polyplex and stained with the PEG antibody show lack of any staining. Scale bar = 50 μm.

With the intention of confirming PanIN specificity and limited off-target uptake, other organs were also examined histologically and by anti-Alexa Fluor 488 and confirmed with anti-PEG immunohistochemical analysis. We were selective in the tissues that we analyzed for immunohistochemistry (IHC), and chose tissues based on ex vivo florescence or likelihood of polyplex accumulation based on the CCK expression pattern or prior knowledge of polyplex distribution. The colon and lung were not selected for both Alexa Fluor 488 and PEG IHC, since these tissues did not demonstrate an ex vivo fluorescent signal, do not have CCK receptors, and historically do not accumulate polyplex. However, we performed Alexa Fluor 488 IHC on lung and PEG IHC on colon to rule out potential polyplex accumulation. As expected, the findings were negative in these tissues. The immunohistochemistry of these other organ tissue sections with the Alexa Fluor 488 antibody was negative but showed some background and positive staining in the kidney tubule (Figure 7A). Immunoreactivity of the excised organs probed with an antibody for PEG (Figure 7B) lacks any staining, and no immunoreactivity was seen in the kidney. This difference in the immunohistochemistry images between the two antibodies suggests that the positive staining identified in the tissues probed with the Alexa Fluor 488 antibody represents staining from the free Alexa Fluor 488, such as after polyplex uptake, degradation, and excretion by the kidney. Slides from the Alexa Fluor 488 immunohistochemistry from three separate mouse pancreata were digitized with an Aperio ScanScope XT (Leica) at 200× in a single z-plane. Cell detection algorithms were run, and the staining intensity was scored using a scale of 0–3 as follows: 0 for no staining, 1 for mild staining, 2 for moderate staining, and 3 for strong staining. The percentage of cells staining positively is reported, and an H-score, [35] which integrates percent positive and staining intensity, was calculated using QuPath [36] as follows:

H-score = [1 × (% cells 1+) + 2 × (% cells 2+) + 3 × (% cells 3+ )]

H-score range = 0–300.

The results of the H-score are shown in Table 1.

These data support the negative ex vivo fluorescence and lack of NP uptake in the other organs visualized in the IVIS. Negative controls were run for each tissue by replacing the primary anti-PEG antibody with a nonspecific antibody reagent from the same host species and isotype (isotype control). Staining was considered specific when there was staining with the primary anti-PEG antibody and no staining in the isotype control or control untreated tissue. The positive and negative anti-PEG-stained control tissues are shown in Supplementary Figure S3. The pancreata from control C57BL/6 wild-type mice treated with CCK-BR-targeted NPs were negative for Alexa Fluor 488 immunoreactivity (Supplementary Figure S4).

### 3.8. Human PanIN Lesions Express CCK-B Receptors

**Figure 8:**
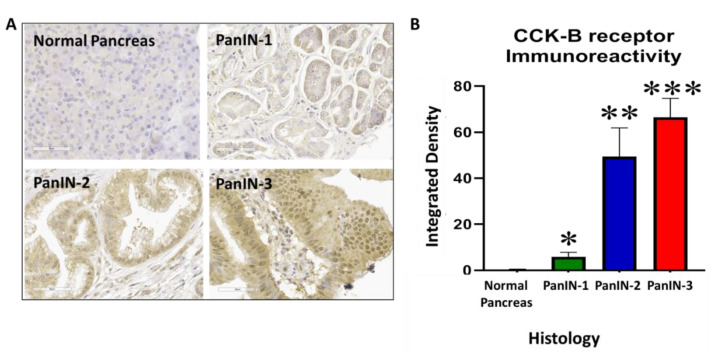
Human pancreas tissue array reacted with the CCK-B receptor antibody. (**A**) A representative image from normal human pancreas tissue and human pancreas tissues with various PanIN grades 1, 2, and 3 is shown. (**B**) Quantitative analysis of all the images from the human pancreas tissue array shows increased CCK-BR immunoreactivity with increasing grade of PanIN lesions. Significantly different from control, normal pancreas tissues: * *p* < 0.05; ** *p* < 0.01; *** *p* < 0.005.

In order to show the clinical relevance and translational potential for use in human subjects, we examined human pancreas tissue using a commercial tissue microarray containing normal pancreas tissues and specimens with PanINs of various grades. This tissue array was stained with the CCK-B receptor antibody (as above in Figure 6E,F), and we demonstrated the absence of CCK-BR immunoreactivity in normal human pancreas (Figure 8A, top left) and the presence of CCK-BR immunoreactivity in human PanINs with increasing grade, including PanIN-1, PanIN-2, and PanIN-3 lesions (Figure 8A). Immunoreactivity for the CCK-BR is negligible in the normal human pancreas, and the staining increases with increasing PanIN grade. Analysis of the CCK-BR immunoreactivity integrated density analyzed by computer software (ImageJ) is plotted for normal human pancreas tissue and increasing PanIN grade (Figure 8B). Integrated density is the sum of the values of the pixels in the image or selection. This is equivalent to the product of area and mean gray value.

## 4. Conclusions

The current recommendations today for pancreatic cancer screening include high-risk individuals with a genetic predisposition or family history of pancreatic cancer [37]. Although endoscopic ultrasound and MRI are used to monitor those subjects with cystic lesions of the pancreas [38], the overwhelming majority of cancers develop from microscopic PanIN lesions that will require more sensitive imaging tools for detection. Although surgical resection offers a potential chance for a cure for pancreatic cancer if detected early [39], over 90% of the subjects have advanced disease at the time of presentation due the absence of sensitive imaging tests and biomarkers [10]. In the present study, we demonstrate the detection of early precancerous lesions (PanINs) in genetically engineered mice. These lesions are not seen by routine radiographic imaging such as MRI, PET, or CT scans. Notably, there is no control PanIN imaging agent available at this time to compare our technology to, which emphasizes the concept’s novelty and utility. In the current investigation, we utilized fluorophores for imaging in a murine model for proof of principle. However, more highly sensitive compounds have been used to enhance imaging in humans such as Fluorine-18 [40], a radiopharmaceutical tracer used for PET (positron emission tomography) imaging, or Technetium-99m (99mTc), used for CT-SPECT imaging [41]. Employing the CCK-BR as a specific target for early detection of precancerous lesions in the pancreas combined with compounds for imaging may increase the number of subjects identified with surgically resectable lesions. Furthermore, if the CCK-BR-targeted NP can also deliver gastrin siRNA [24] or other payloads to the PanIN lesions such as siRNA for mutant KRAS, these NPs would have the potential not only to identify but also to also treat these PanIN lesions to decrease proliferation and halt the progression to cancer. 

## 5. Patents

Georgetown University and the NIH hold intellectual property for this work.

## Figures and Tables

**Table 1:** Alexa Fluor 488 immunoreactivity scores in tissues. Three separate 5-month-old mouse organs were scored for positive Alex-488 antibody staining. The percentage of immunoreactive cells per tissue and the H-score show only increased staining in the PanIN lesions of the pancreas, while the normal pancreas and other major organs are negative.

	Normal Pancreas		PanINs		Liver		Kidney		Heart		Stomach	
Sample	% positive	H- Score	% positive	H- Score	% positive	H- Score	% positive	H- Score	% positive	H- Score	% positive	H- Score
ID98	0.01	0.01	9.33	21.82	0	0	0.02	0.02	0.06	0.07	0	0
ID101	0	0	18.48	31.39	0.02	0.02	0.07	0.13	0	0.01	0	0
ID114	0	0	31.72	58.32	0.01	0.01	0.01	0.01	0	0	0	0

## Data Availability

The data presented in this study are available on request from the corresponding author. The data are not publicly available due to pending intellectual property.

## Supplementary Materials

The following are available online at https://www.mdpi.com/article/10.3390/biom11121766/s1: Figure S1: Thiol-Maleimide reaction, Figure S2: In vivo imaging of a KC mouse after injection with untargeted NPs, Figure S3: Title: Immunohistochemistry controls. Figure S4: Title: Negative control mouse pancreas immunohistochemistry.
