# Supplementary material for: Cholecystokinin-B Receptor-Targeted Nanoparticle for Imaging and Detection of Precancerous Lesions in the Pancreas
Source: Biomolecules. 2021 Nov 25;11(12):1766. doi: 10.3390/biom11121766 (PMC8698999; doi:10.3390/biom11121766)
Supplement: Supplementary file 1 [file biomolecules-11-01766-s001.zip › biomolecules-1455175-supplementary.pdf]

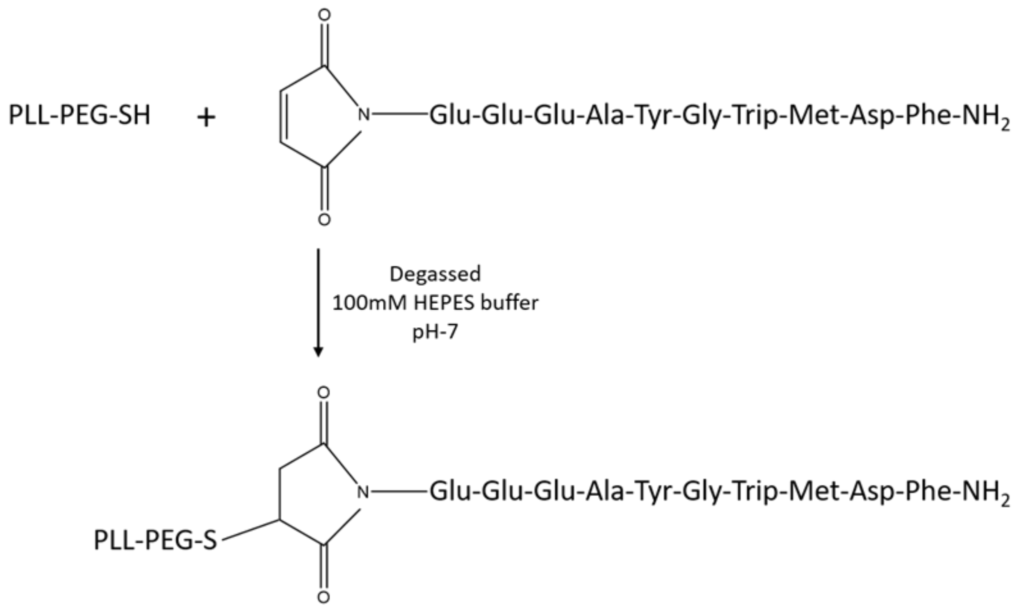

Figure S1. Thiol-maleimide reaction. Maleimide functionalized gastrin-10 peptide reacts with thiol group of the polymer and forms a stable thioether bond. From pH 6.5 to 7.5, the thiol-maleimide reaction is chemoselective for thiols. At pH 7.0, the reaction rate of thiol with maleimide is about 1000 times faster than the reaction rate for amines with maleimide.

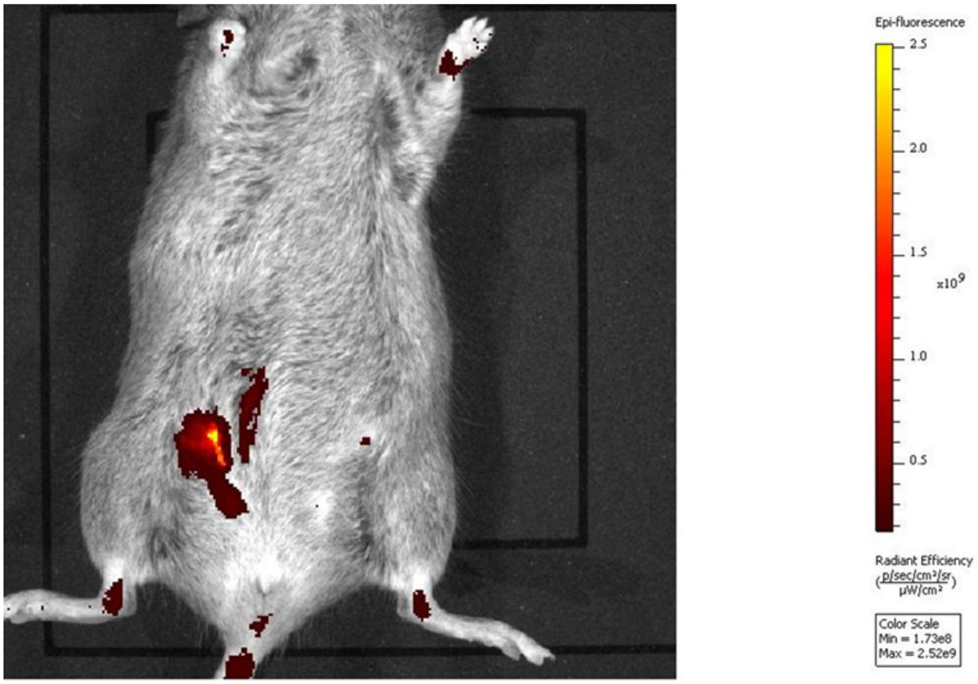

Figure S2. *In vivo* imaging of a KC mouse after injection with untargeted NPs. Five month old KC mouse injected with untargeted NPs shows no specific fluorescence in the mouse pancreas after 5 hours in IVIS imaging machine and some residual NP at the site of IP injection.

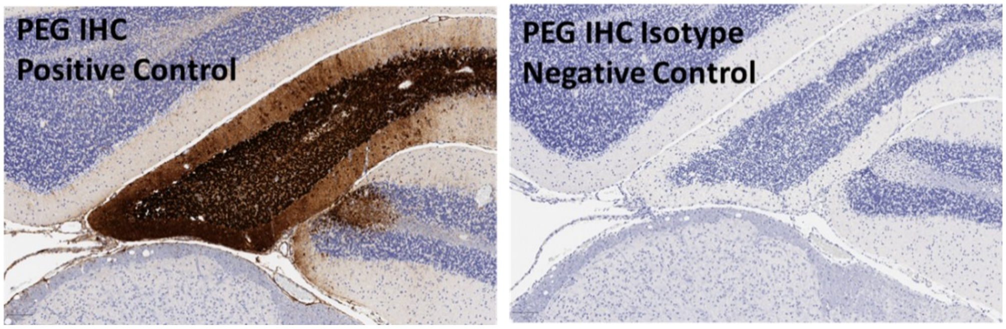

Figure S3. Immunohistochemistry controls. Immunohistochemistry positive (left) and negative (right) controls for PEG are shown.

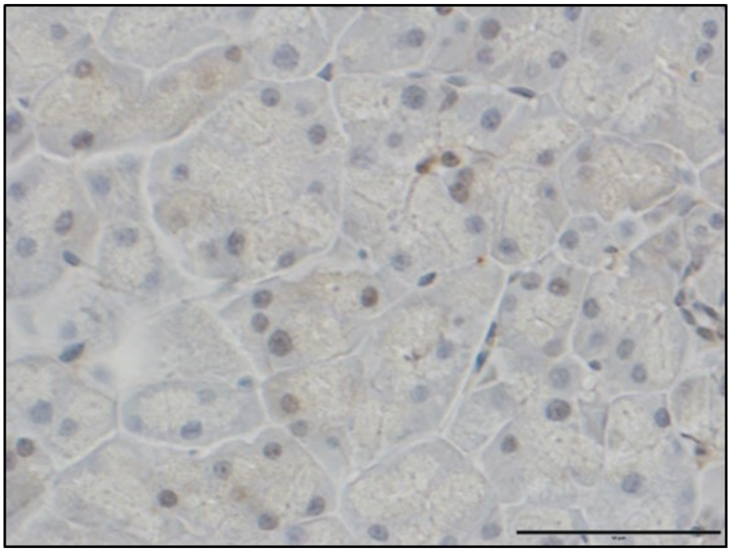

Figure S4. Negative control mouse pancreas immunohistochemistry. Wild-type C57BL/6 mice were injected with targeted fluorescent polyplex nanoparticles and the pancreas excised and reacted by with anti-Alexa488. No significant immunoreactivity was identified. Bar = 200µm.
